# Mechanical process prior to cryopreservation of lipoaspirates maintains extracellular matrix integrity and cell viability: evaluation of the retention and regenerative potential of cryopreserved fat-derived product after fat grafting

**DOI:** 10.1186/s13287-019-1395-6

**Published:** 2019-09-23

**Authors:** Jingwei Feng, Wansheng Hu, Mimi Lalrimawii Fanai, Shengqian Zhu, Jing Wang, Junrong Cai, Feng Lu

**Affiliations:** Department of Plastic and Reconstructive Surgery, Nanfang Hospital, Southern Medical University, Guangzhou, 510515 China

**Keywords:** Cryopreservation, Stromal vascular fraction gel, Fat grafting, Wound healing

## Abstract

**Background:**

Cryopreservation of fat grafts facilitates reinjection for later use. However, low temperature and thawing can disrupt tissues and cause lipid leakage, which raises safety concerns. Here, we compared the cryopreservation potential of stromal vascular fraction (SVF) gel processed from lipoaspirate with that of fat.

**Methods:**

Human SVF gel and fat were cryopreserved at − 20 °C without cryoprotectant for 1 month. Fresh SVF gel and fat were used as controls. Tissue viability, adipose-derived stem cell (ASC) function, and the extracellular content were evaluated. At 3 months after transplanting the specimens to immunocompromised mice subcutaneously, the grafts were examined for retention, tissue engraftment, and inflammatory levels. The regenerative effect of cryopreserved SVF gel was evaluated in a murine ischemic wound healing model.

**Results:**

At 1 month, the cell death rate in the SVF gel group was 36 ± 2%. The survived ASCs not only could be isolated via explant culture but also preserved colony-forming and differentiation. However, prolonged cryopreservation exacerbated apoptosis. Assessment of recovered tissues showed that the morphology, cell viability, and extracellular protein enrichment were better in SVF gel-preserved tissues than in frozen fat. At 3 months after lipotransfer, the retention ability of 1-month cryopreserved fat was 41.1 ± 9% compared to that of 1-month cryopreserved SVF gel. Immunostaining results showed that adipose tissue regeneration and integrity in the 1-month cryopreserved SVF gel group were superior to those of the cryopreserved fat group. The cryopreserved SVF gel also accelerated healing of the ischemic wound, compared with cryopreserved fat.

**Conclusion:**

Cryopreserved SVF gel maintained tissue integrity and cell viability and resulted in a better long-term retention rate than that of cryopreserved fat. Cryopreserved SVF gel also showed superior regenerative potential and improved ischemic wound healing.

## Background

Autologous fat grafting has significant therapeutic potential in revolumization and regenerative medicine [1]. Although the techniques for autologous fat transplantation have been optimized, they are associated with variable tissue resorption rates, requiring multiple surgeries to maintain the desired therapeutic effect [2, 3]. This limitation increased the scientific and clinical interest in the process of adipose tissue cryopreservation for reconstructive or regenerative medical purposes [4, 5]. However, current cryopreservation techniques remain controversial, and evidence supporting their use is insufficient.

Although cryopreservation of fat tissue has been studied extensively, the results remain controversial [6–8]. In addition to the impracticality of the highly demanding freezing procedure, cryopreservation of fat tissue can induce substantial fat necrosis and may lead to complications such as oil cysts, fat granuloma, and chronic inflammation [9, 10]. Adipocytes are vulnerable under stress conditions such as exposure to ischemia and freezing [11, 12]. Transplantation of cryopreserved fat tissue can lead to the formation of oil cysts. Adipose-derived stem/progenitor cells (ASCs) are viable after cryopreservation and more likely to survive the hypoxic insult after transplantation [13]. Therefore, we hypothesized that removing adipocytes from fat tissue before cryopreservation may improve its tolerance during the cooling and recovery procedures, thereby increasing the viability of fat tissue and its regenerative potential.

We previously reported a simple mechanical process to selectively eliminate most mature adipocytes within the adipose tissue [14]. Briefly, this is achieved by shifting the lipoaspirate between two syringes. The application of an adequate shear stress to the tissue eliminates adipocytes without affecting stem cells, which are protected by their smaller size. The procedure results in a product known as stromal vascular fraction (SVF) gel, which is particularly rich in ASCs and native extracellular matrix (ECM). SVF gel transplantation results in a unique regeneration process and a better retention rate than that of traditional fat grafting in a mouse model [15]. In a clinical study conducted by our group, SVF gel transplantation resulted in a better long-term graft retention rate and greater patient satisfaction than traditional fat grafting [16]. Moreover, SVF gel shows great potential in regenerative medicine, as it improves ischemic flap survival, remodels hypertrophic scars, and accelerates wound healing [17–19].

In this study, we tested SVF gel cryopreservation in a mouse model. The tissue viability of cryopreserved SVF gel was compared with that of cryopreserved fat tissue, and the fate of the grafted frozen SVF gel and its regenerative effect were assessed in a wound healing model.

## Materials and methods

### Human SVF gel preparation

**Fig. 1:**
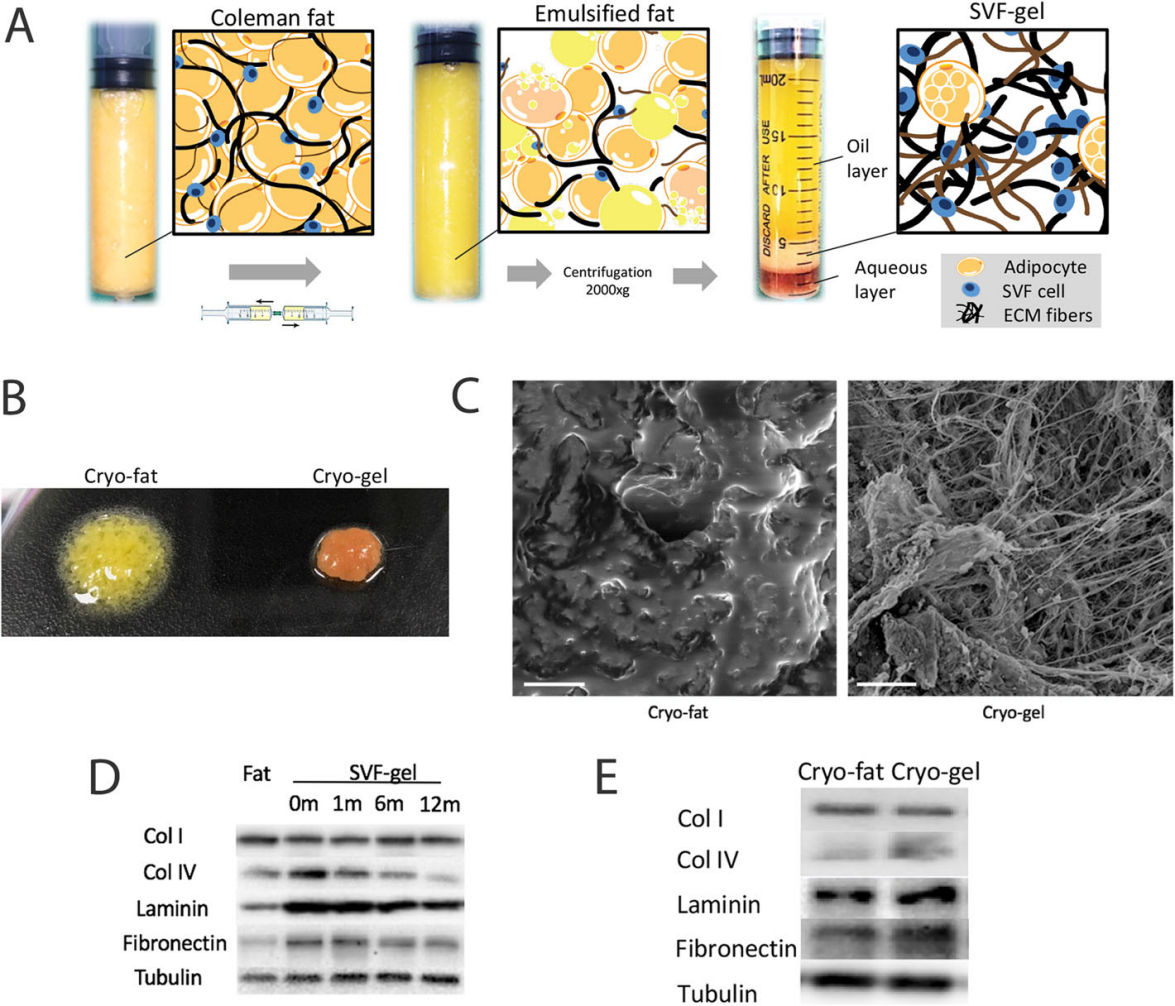
Characterization of the SVF gel. **a** Schematic diagram of SVF gel preparation. Lipoaspirates were centrifuged (1200×*g*, 3 min) to prepare Coleman fat (dense but intact fat without tumescent fluid and blood), which was transferred into a secured SVF gel Luer-lock syringe and passed through the syringe for shearing. The content of the syringe was centrifuged, and the resulting pellet was termed “SVF gel”. **b** Gross appearance. After cryopreservation for 1 month and thawing for 1 min, the cryo-fat pellet collapsed at room temperature and became covered by the released oil. However, the cryo-gel pellet maintained its shape. **c** Low-magnification scanning electron microscope (SEM) images. The cryopreserved SVF gel was enriched in ECM content, whereas the cryo-fat was immersed in oil and no clear micro-structure was observed. Scale bar = 100 μm. **d** Western blotting. The extracellular content was measured and compared between fresh fat and the SVF gel. The SVF gel contained a greater amount of ECM protein than the fat aspirate. However, a gradual loss of ECM protein (or antigen function) was observed in Col IV and fibronectin, but not in Col I. **e** Western blotting. The extracellular content was measured and compared between 1-month cryo-fat and 1-month cryo-gel. The cryo-gel contained a greater amount of Col IV and fibronectin than cryo-fat. *n* = 3 independent experiments with the fat of 3 donors

This study was approved by the Ethics Committee of Southern Medical University, Nanfang Hospital. Lipoaspirate was obtained from eight non-obese healthy female human donors who underwent liposuction at ages ranging from 23 to 51. Abdominal or femoral fat was harvested using the tumescent technique by infiltrating with saline (1:1,000,000 epinephrine), and subcutaneous fat was suctioned manually using a 20-cc syringe equipped with a 3.0-mm cannula. SVF gel was prepared as previously described [14]. Briefly, the lipoaspirate was centrifuged at 1200×*g* for 3 min to obtain “Coleman fat” in the middle layer. The bottom layer of the tumescent fluid and the top oil layer were discarded, and 20 cc of Coleman fat was transferred to two 20-cc syringes connected by an SVF gel Luer-lock connector. The Coleman fat was then transferred between two syringes (6–8 times) at a rate of 20 mL/s until the Coleman fat was converted into a uniform emulsion, which was then centrifuged at 2000×*g* for 3 min. The middle layer, which had a volume of 15–20% of the original Coleman fat, was identified as SVF gel and collected for further use (Fig. 1a).

### SVF gel cryopreservation and recovery

Freshly prepared SVF gel or Coleman fat (lipoaspirate condensed by centrifugation at 1200×*g* for 3 min) in an aseptic packaging was directly transferred into a − 20 °C freezer and incubated for 1–3 months. The cryopreserved fat or SVF gel was thawed by soaking in a 37 °C water bath for 3 min.

### Morphologic assessment by a scanning electron microscope

Samples were fixed with 2% glutaraldehyde in 0.1 M phosphate buffer, post-fixed in 1% osmium tetroxide in the same buffer for 1 h, dehydrated in increasing concentrations of acetone, and critical-point dried. Stubs were fixed to the samples with colloidal silver, and the samples were sputtered with gold using a MED 010 coater and examined with an S-3000N scanning electron microscope (HITACHI company, Japan). Samples from three donors were imaged, and representative photos were demonstrated.

### TUNEL assay and quantification

Fresh lipoaspirate, fresh SVF gel, and cryopreserved fat or SVF gel frozen for 1 month or 3 months were prepared for 20-μm cryosections. Each experiment’s samples were from the same donor. Samples were snap-frozen and stored in − 80 °C before lysis. The sections were fixed at room temperature and washed with phosphate-buffered saline (PBS) for 30 min, and with 0.1% Triton-X for 2 min on ice. The TUNEL assay was performed using an in situ cell death detection kit following the manufacturer’s instructions (Roche, Basel, Switzerland) with proper controls. The cell nucleus was labeled with Hoechst 33342 (Thermo Fisher Scientific, Waltham, MA, USA). Whole nuclei and TUNEL+ nuclei were quantified from a 300 × 300 μm field of each cryo-section. Values were expressed as the mean ± SD from three independent samples/experiments.

### Colony-forming assay

ASCs were isolated by explant culture with fresh fat, fresh SVF gel (generated from 1 cc of fat), cryo-fat, or cryo-gel preserved for 1 month or 3 months. Each 0.1-cc tissue sample was scratched onto the surface of a 10-cm tissue-culture dish using syringe needles and cultured for 14 days in full-growth DMEM/F12 medium (supplemented with 10% fetal bovine serum) for primary culture. One half of the medium was carefully replaced every other day without disturbing the attached tissue. On day 14, 5 × 10^3^ ASCs were isolated from approximately 0.3 cc of fresh fat, 0.05 cc of fresh SVF gel, 0.13 cc of cryo-gel, and 1.2 cc of cryo-fat and seeded onto different 3.5-mm culture dishes. Dishes were incubated with full-growth medium, which was replaced every 3 days. On day 7 of culture, dishes were air dried, fixed with methanol for 5 min, and stained with 4% Giemsa solution for 5 min. Colonies larger than 0.5 mm in diameter were counted under a light microscope using digital imaging software (Photoshop; Adobe Systems Inc., San Jose, CA, USA). The assay was performed using three replicates from three different donors.

### Multilineage differentiation assay

A total of 2 × 10^5^ cells from the 2nd passage acquired from the explant culture were seeded and cultured until reaching confluency. Differentiation into adipogenic, osteogenic, and chondrogenic lineages was performed under the following conditions: for adipogenesis, ASCs were incubated for 15 days in DMEM with 10% FBS, 0.5 mM isobutyl-methyl-xanthine, 1 M dexamethasone, 10 μM insulin, and 200 μM indomethacin; for osteogenesis, ASCs were incubated for 15 days in DMEM with 10% FBS, 0.1 mM dexamethasone, 50 mM ascorbate-2-phosphate, and 10 mM glycerophosphate; and for chondrogenesis, ASCs were incubated for 15 days in DMEM containing 1% FBS, 6.25 mg/mL insulin, 10 ng/mL TGFβ-1, and 50 nM ascorbate-2-phosphate. The differentiation medium was changed every 3 days and cells were cultured for 21 days in 5% CO_2_ at 37 °C. Then, the three lineages were analyzed qualitatively using Nile red (adipogenic), von Kossa (osteogenic), and Alcian blue (chondrogenic) staining. Differentiation assay were conducted in triplicate using ASCs of one donor.

### Western blot analysis

Western blotting was conducted in triplicate, and each experiment’s samples were from the same donor. Fresh or frozen tissue was snap-frozen in liquid nitrogen and stored in − 80 °C before lysis. Then samples were lysed using cold RIPA buffer (Pierce, MA, USA) following homogenization with an electric homogenizer. The samples were then centrifuged, and the supernatant was collected. Samples were separated by electrophoresis under reducing and denaturing conditions, transferred to a nitrocellulose membrane, and immunoblotted using COL1A1 (3G3) monoclonal antibody (LifeSpan BioSciences, WA, USA), mouse anti-collagen IV antibody clone MC4-HA (United States Biological, MA, USA), and monoclonal anti-laminin antibody (Biorbyt, CA, USA). Western blots were developed using the ECL substrate.

### Fat grafting in an immunodeficient mouse model

Animal care and treatment were performed according to institutional guidelines. The experimental protocol was approved by the institutional review board. Ten-week-old female nude mice (BALB/cAJcl-FOXN1nu/nu) weighing 20–23 g were purchased from Southern Medical University Experimental Animal Center (Guangzhou, China). After undergoing general anesthesia with 1% sodium pentobarbital (50 mg/kg), 36 mice (*n* = 6 mice for each group) were injected subcutaneously into the dorsal area with 0.3 mL of fresh fat, fresh SVF gel, 1- or 3-month cryopreserved fat (cryo-fat 1 month or 3 months), or 1- or 3-month cryopreserved SVF gel (cryo-gel 1 month or 3 months) from 1 donor. At 3 months postoperatively, grafts were harvested, weighed, and fixed.

### Flow cytometry

Grafts from the fresh SVF gel group, 1-month-frozen SVF gel group, fresh fat group, and 1-month-frozen fat group were harvested at 1 month after transplantation. Samples were digested, and the SVF was assessed by flow cytometry. Briefly, 0.5 cc of graft tissues was minced with scissors and incubated in 0.075% collagenase buffer in a shaking water bath (37 °C, 30 min) for digestion. After neutralization and filtration, SVF cells were washed and stained with the following antibodies and corresponding isotype controls: anti-CD11b-PE (Miltenyi Biotec, Bergisch Gladbach, Germany), anti-F4/80-APC (eBioscience, Inc., CA, USA), rat IgG2a Control APC (BD Biosciences), and rat IgG2a Kappa Control PE (BD Biosciences). Samples were analyzed by flow cytometry (MACSQuant Analyzer 10; Miltenyi Biotec). Gating for each signal was set to eliminate 99.9% of the cells in the corresponding isotype control. Experiments were conducted in triplicate using 0.5-cc grafts collected from three mice in the same groups.

### Ischemic wound healing model

Eighteen female nude mice (*n* = 6 mice for each group) were used to establish an ischemic wound healing model as previously reported [20]. Briefly, one round full-thickness wound (5-mm radius) extending through the panniculus carnosus was made on the dorsum. A bipedicled flap measuring 3.0 cm in length and 3.0 cm in width was generated around the wound. The 18 nude mice were divided into the control group (receiving 0.1 mL of PBS), the cryo-fat group (receiving 0.1 mL of cryo-fat 1 month), and the cryo-gel group (receiving 0.1 mL of cryo-gel 1 month). On day 0, fat-derived products from the same donor or PBS were injected to the wound. Wounds were then dressed with Tegaderm sterile dressing (3M Healthcare, St Paul, MN, USA), which was changed every other day until wound closure or until day 14. Digital photographs were taken at the time of surgery and at 2, 5, 8, 10, and 14 days after surgery. The wound area was quantified using Photoshop.

### Histology and immunofluorescence staining

Samples were embedded in paraffin, sectioned, and stained with hematoxylin and eosin (H&E) and Masson’s trichrome (MT). Staining was performed according to standard protocols. Different sections were examined under a light microscope (Olympus BX51) and evaluated. Immunofluorescence staining of 5-μm-thick sections of the harvested tissue samples was performed with the following primary antibodies: Guinea pig anti-Perilipin/PLIN1 (Progen, Heidelberg, Germany) and rabbit anti-von Willebrand factor (DAKO). Samples were incubated in primary antibody solution for 16 h at 4 °C, followed by secondary antibodies with Hoechst in TBST for 1 h and mounting.

### Statistical analysis

Statistical analyses were performed using the SPSS statistical software program 20.0 (SPSS, Inc., Chicago, IL, USA). The results were presented as the mean ± SD. Data were analyzed with the non-parametric Kruskal-Wallis rank sum test followed by Dunn’s post hoc test for group comparisons. *p* values < 0.05 were considered statistically significant.

## Results

### The extracellular matrix is preserved in SVF gel after cryopreservation

After cryopreservation for 1 month and thawing for 3 min, 0.1 mL of cryo-gel and cryo-fat were injected into a dish. After 1 min, the cryo-gel retained its original shape, whereas the cryo-fat pellet collapsed and released transparent oil droplets within the tissue (Fig. 1b). Scanning electron microscope (SEM) results showed that ECM fibers were preserved in the cryo-gel, whereas the cryo-fat was immersed in oil and no clear micro-structure was observed (Fig. 1c). ECM protein density was examined by western blotting. Before cryopreservation, the SVF gel contained a greater amount of ECM protein than the lipoaspirate because of tissue compression and the oil removal process. However, a gradual loss of collagen (Col) IV and fibronectin was observed after cryopreservation, whereas the more abundant adipose matrix Col I remained intact (Fig. 1d). One-month cryo-gel had a greater amount of Col IV and fibronectin than 1-month cryo-fat (Fig. 1e).

### SVF gel decreases cryopreservation-related cell death

**Fig. 2:**
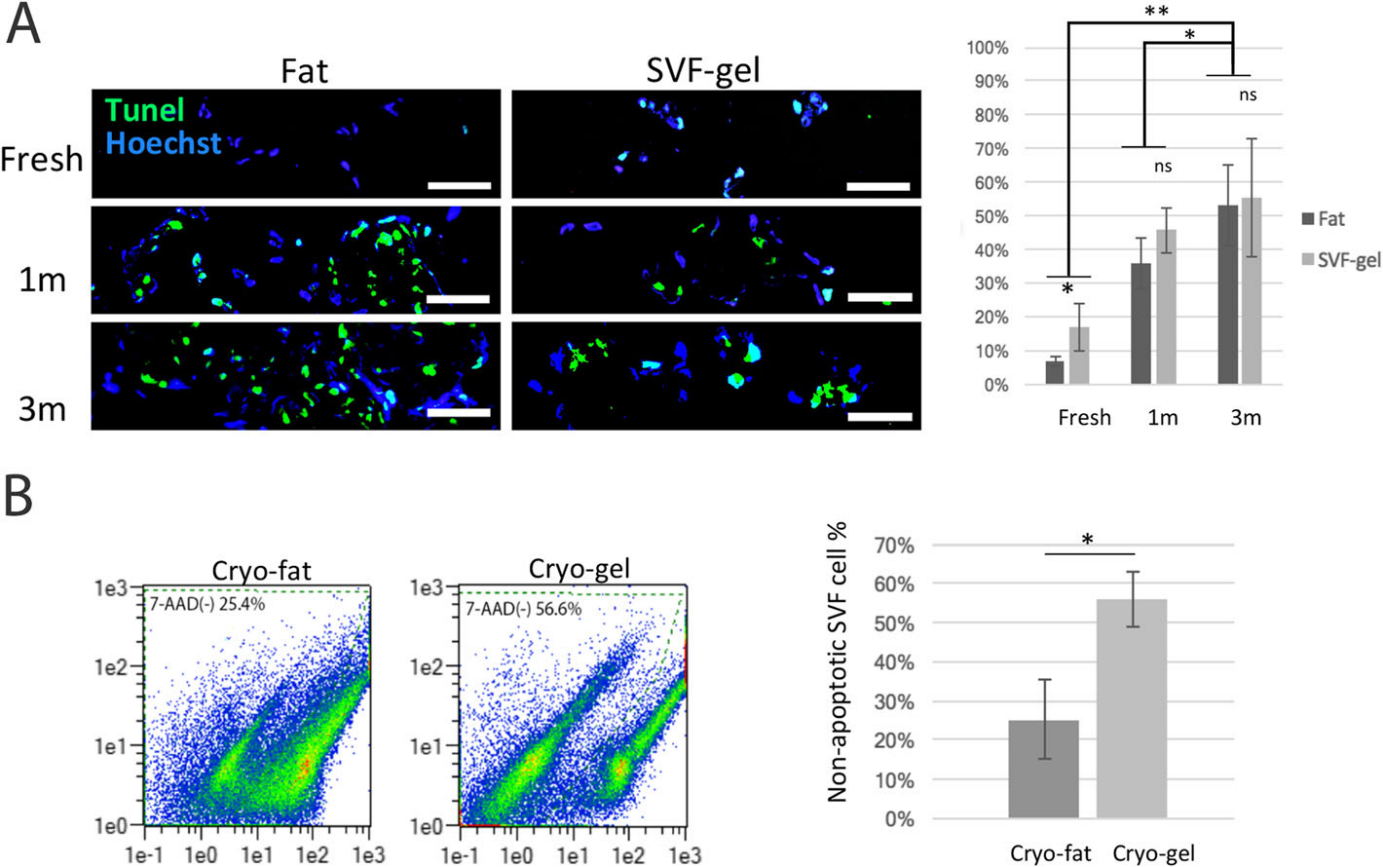
Freeze-induced tissue injury. **a** TUNEL assay of tissue cryosections. Frozen sections of fresh fat, fresh SVF gel, and 1- or 3-month cryopreserved fat and SVF gel were subjected to the TUNEL assay to measure apoptosis (TUNEL+ cells). Prolonged cryopreservation without heat recovery exacerbated SVF cell apoptosis. The rate of SVF cell death was 36 ± 1% at 1 month and 53 ± 3% after 3 months of freezing. **p < 0.01. Scale bar = 50 μm. **b** Flow cytometry density plot of 7-AAD staining for SVF cells. SVF cells collected by enzymatic digestion were analyzed by flow cytometry. Non-apoptotic cells were 7-AAD-negative. The 7-AAD (−) population is enclosed within the green dash line. The percentage of non-apoptotic cells was more than twofold greater in cryo-gel (56 ± 7%) than in cryo-fat (25 ± 1%). *p < 0.05. *n* = 3 independent experiments with 3 donors

The frozen SVF gel or fat was examined before thawing using TUNEL staining to measure freeze-induced cell death compared with that in fresh SVF gel or fat (Fig. 2a). Fresh SVF gel had more apoptotic cells (16 ± 17%) than fresh fat (6.81 ± 1.5%) because of mechanical manipulation. Freezing at − 20 °C induced apoptosis, and prolonged cryopreservation exacerbated apoptosis (*p* = 0.0121). In SVF cells, the rate of apoptosis was 36 ± 1% at 1 month and 53 ± 8% after 3 months. However, cell viability did not differ significantly between cryo-fat and cryo-gel. Cell death after thawing was also tested using flow cytometry (Fig. 2b). Enzymatic digestion of the frozen tissue showed that the rate of non-apoptotic cells (7-AAD-negative) in cryo-fat (25 ± 1%) was less than half of that in cryo-gel (56 ± 7%). The TUNEL assay showed that cell viability before thawing was similar between cryo-fat and cryo-gel, whereas after thawing, cell viability was significantly better in the cryo-gel (*p = 0.0121).

### SVF gel preserves cellular viability after cryopreservation

**Fig. 3:**
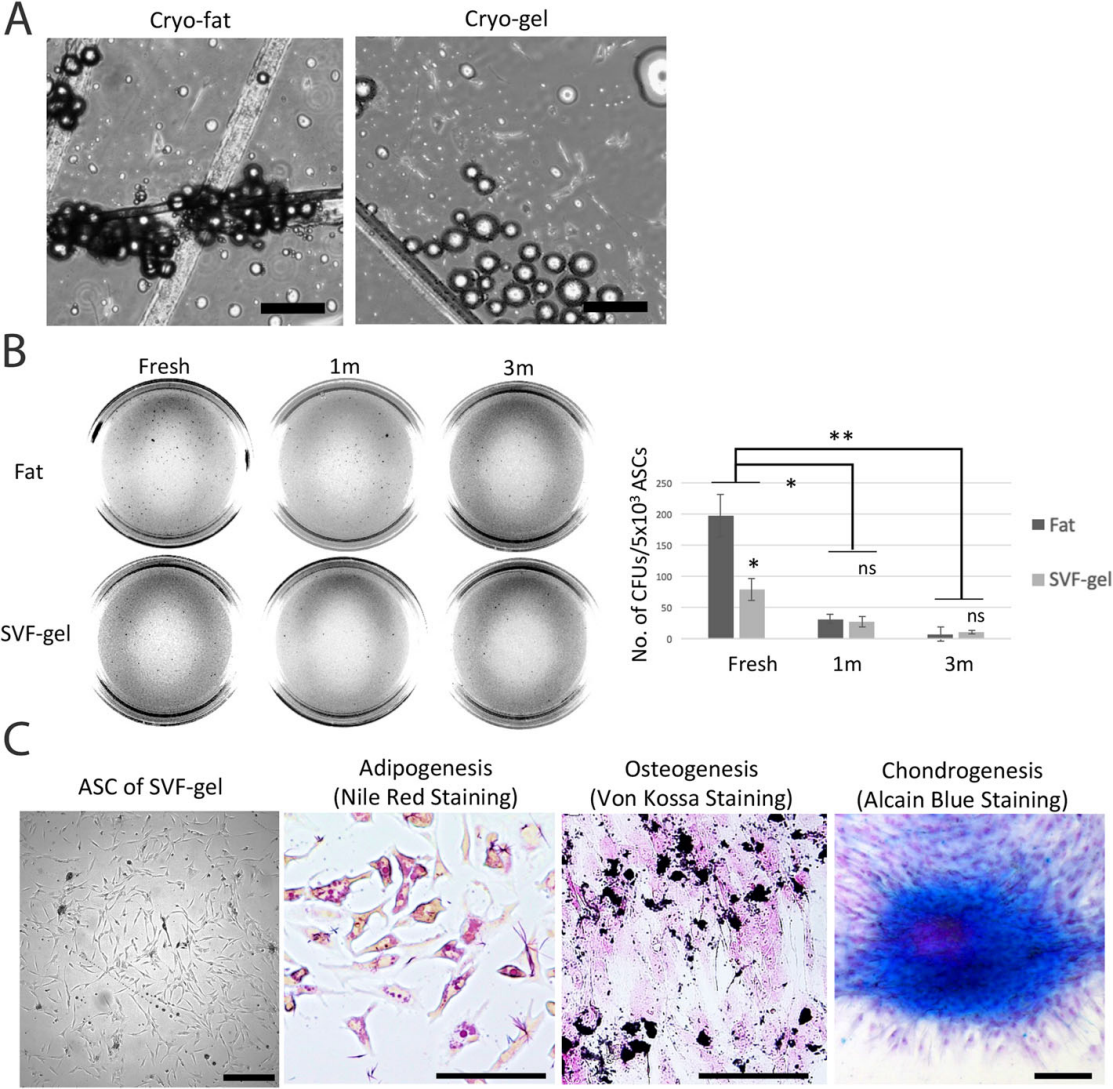
Cell viability after cryopreservation. **a** Explant culture of ASCs isolated from recovered cryo-fat and cryo-gel. Scale = 100 μm. **b** Colony-forming assay. Viable ASCs from SVF gel grew significantly fewer colonies than those from fresh fat. Short-term freezing (1 month) impaired the colony-forming ability of ASCs. *p = 0.0186, **p = 0.0074. *n* = 3 independent experiments with 3 donors. **c** Multilineage differentiated assay. ASCs from 1-month cryopreserved SVF gel maintained the adipogenic, osteogenic, and chondrogenic differentiation potential. The adipogenic group showed pink Nile Red (+) oil droplets. The osteogenic and chondrogenic groups showed positive staining for calcium deposition (black) or glycosaminoglycans (blue); the nucleus is stained in pink. Scale bar = 100 μm. *n* = 3 replicate with 1 donor

The explant culture of the cryopreserved tissue after thawing showed that fibroblast-like cells migrated from both cryo-fat and cryo-gel by day 14 (Fig. 3a). The colony-forming ability of ASCs from cryopreserved tissues was also tested. Viable ASCs present in cryo-fat or cryo-gel migrated to the dish surface and were isolated by explant culture. Viable ASCs from the SVF gel produced significantly fewer colonies than those from fresh fat. Short-term freezing for 1 month impaired the colony-forming ability of ASCs (*p = 0.0186). ASCs from the SVF gel cryopreserved for 1 month expanded easily in vitro and showed fibroblast-like morphology (Fig. 3b). To verify their multipotency, ASCs were incubated in specific media to induce differentiation into adipogenic or osteogenic lineages. Adipogenic differentiation was determined by Oil-Red-O staining of intracellular lipid droplets, and osteogenic differentiation was confirmed by Alizarin red staining of matrix mineralization. ASCs were stained with Alcian blue to examine the chondrogenic differentiation potential (Fig. 3c).

### Cryo-gel grafting has a better long-term retention rate than cryo-fat

**Fig. 4:**
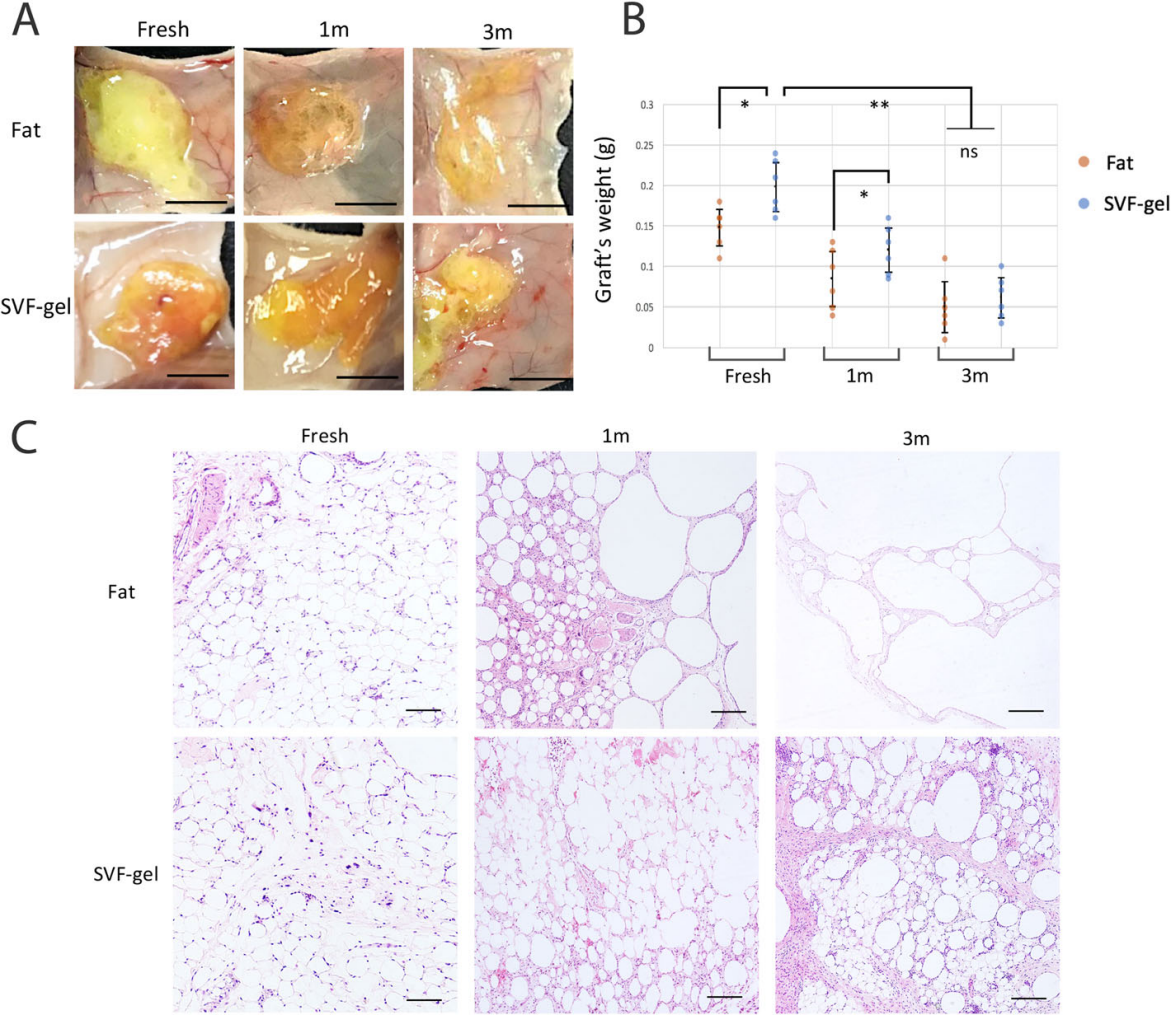
Fat grafting into an immunodeficient mouse model. **a** Gross appearance of the representative grafts. Cryopreserved human fat or SVF gel was transplanted subcutaneously into mice, and grafts were harvested after 3 months. Scale bar = 1.0 cm. **b** Weight retention. The SVF gel showed superior weight retention over the fat groups. Extended cryopreservation decreased the cryo-gel graft retention rate from 41.1 ± 0.141% (1-month group) to 24.1 ± 8% (3-month group), *p = 0.019*,* **p_cryo-fat 1 month vs. cryo-gel 1 month_ = 0.016. The retention rate of the 1-month cryo-fat graft decreased markedly, barely reached one half of the retention of the 1-month cryo-gel (16.9 ± 0.077%). *n* = 6 mice. **c** H&E staining. The cryo-fat (1 month) graft had more oil droplets than the cryo-gel (1 month) graft. Cryo-fat (3 months) showed severe fat necrosis and increased oil cyst formation

Fresh fat, SVF gel, and 1-month and 3-month cryopreserved fat or SVF gel were transplanted into the subcutaneous tissue of nude mice. Grafts were harvested and analyzed at 3 months postoperatively (Fig. 4a). The SVF gel group showed better weight retention than the fat group. After prolonged freezing periods, the cryo-gel graft retention rate decreased from 41 ± 14% (1 month) to 24 ± 8% (3 months) (*p = 0.019; **p_cryo-fat 1 month_ vs. _cryo-gel 1 month_ = 0.016). The retention rate of the cryo-fat 1 month graft decreased markedly to less than half of the retention of cryo-gel 1 month (17 ± 8%) (Fig. 4b). Histological analysis showed that oil cyst formation was lowest in samples from the fresh SVF gel or fat group, whereas the 1-month cryo-gel graft had fewer oil cysts and better tissue integrity than the 1-month cryo-fat. However, the 3-month cryo-fat graft was composed of large oil cysts, and the normal adipose tissue structure was not maintained. The 3-month cryo-gel graft showed fewer oil cysts than the 3-month cryo-fat and was characterized by severe inflammation and many small oil cysts (Fig. 4c).

### The cryo-gel graft shows decreased inflammatory cell infiltration and superior adipose tissue structure

**Fig. 5:**
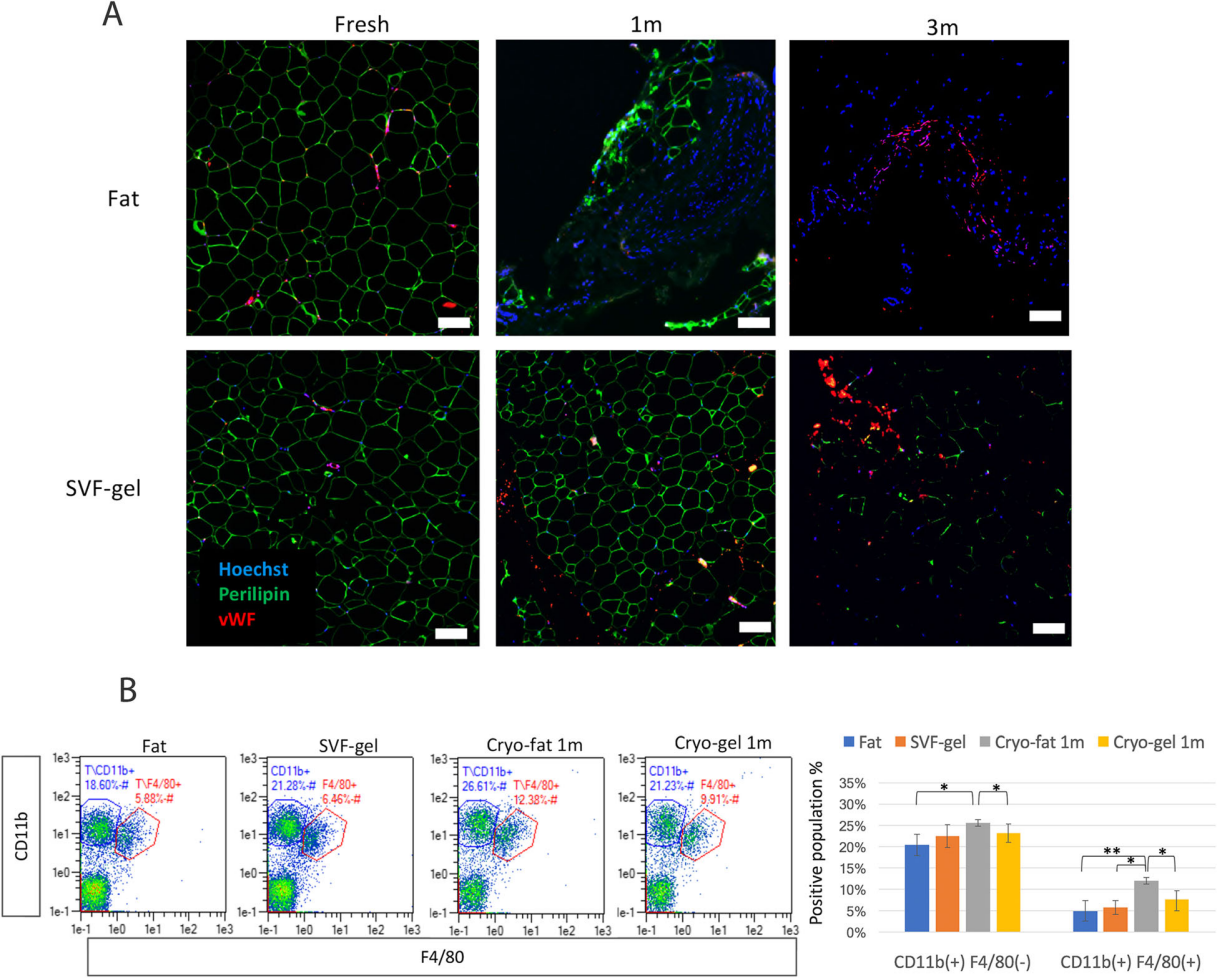
Histological evaluation of the grafts. **a** Immunofluorescent staining. Viable adipocytes were labeled with Perilipin, and vasculature was labeled with vWF. Fresh fat and SVF gel grafts showed Perilipin+ mature adipose tissue. The 1-month cryo-gel, but not the 1-month cryo-fat, contained healthy adipose tissue. Adipogenesis was reduced in tissues frozen for 3 months. **b** FACS analysis of inflammatory cells. CD11b and F4/80 were used as pan-macrophage markers, and CD11b+ F4/80− cells were considered macrophage-depleted leukocytes. The fresh fat and SVF gel grafts had the lowest percentages of macrophage infiltration (5.88% and 6.46%, respectively), whereas the 1-month cryo-fat graft showed increased inflammation, with 12.38% of macrophages among SVF cells. After 3 months, the cryo-fat (1 month) graft showed prolonged macrophage activation, indicating chronic adipose tissue inflammation. *n* = 3 replicate

The cryo-gel (1 month) graft maintained the adipose tissue structure with uniform Perilipin+ living adipocytes, whereas cryo-gel (3 months) and cryo-fat (1 and 3 months) had few Perilipin+ living adipocytes (Fig. 5a). Chronic inflammatory cell infiltration was examined in collagenase-digested grafts (Fig. 5b). CD11b and F4/80 were used as pan-macrophage markers. CD11b+ F4/80− cells are considered a macrophage-depleted leukocyte population including neutrophils, natural killer cells, and granulocytes. Fresh fat and the SVF gel graft had the lowest percentages of macrophages (5.03 ± 2.42% and 6.4 ± 1.64%, respectively) and CD11b+ leukocytes (20.47 ± 2.49% and 22.53 ± 2.67%), whereas the 1-month cryo-fat graft showed a higher degree of inflammation, as 12.23 ± 0.76% of the SVF cells were macrophages. After 3 months, the cryo-fat (1 month) graft showed prolonged macrophage activation indicating chronic adipose inflammation.

### The cryo-gel accelerates the ischemic wound healing process

**Fig. 6:**
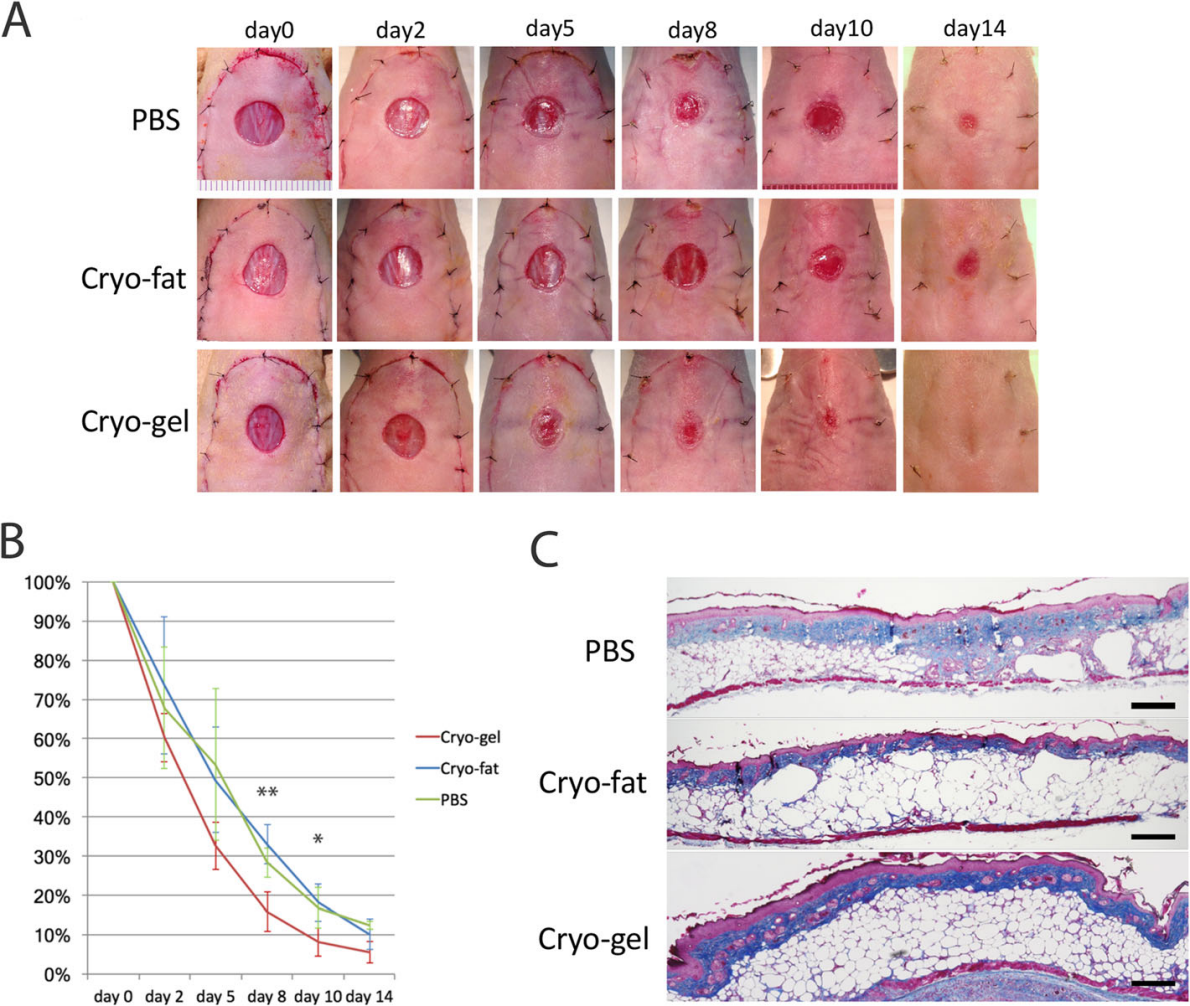
Regenerative potential of the cryo-gel in an ischemic wound healing model. **a** Gross appearance. PBS (control group), cryo-fat, or cryo-gel (0.1 cc each) was injected around the wound periphery. The cryo-gel accelerated the ischemic wound healing process, which was not observed with the cryo-fat. **b** Measurement of wound size. On day 8, the wound size in the cryo-gel group was significantly smaller than that in the other two groups, and a significant difference was still observed on day 10. The cryo-gel group was the first to show wound healing on day 14, which was not observed in the other two groups. **c** H&E staining. On day 14, the skin of the cryo-gel group had a thicker fat layer and complete skin appendages, whereas fat necrosis and thickened interstitial fibrous tissue were observed in the cutaneous fat layer in the other groups. *n* = 6 mice

The gross appearance of the ischemic wound indicated that the cryo-gel accelerated the ischemic wound healing process, which was not observed with the cryo-fat (Fig. 6a). On day 8, the wound size was significantly smaller in the cryo-gel group than in the other two groups (*p* = 0.0375;**p gel vs. con, *p* gel vs. fat < 0.05), and a significant difference was still observed on day 10 (*p* = 0.4677; *p* gel vs. fat < 0.05). The cryo-gel group was the first to show wound healing on day 14, which was not observed in the other two groups (Fig. 6b). H&E staining showed that on day 14, the skin of the cryo-gel group had a thicker fat layer and complete skin appendages. In the other two groups, fat necrosis and thickened interstitial fibrous tissue were observed in the cutaneous fat layer (Fig. 6c).

## Discussion

Regenerative medicine is an alternative strategy for the treatment of various diseases. Cryopreservation is a practical method for expanding the source of autologous cells. Methods for the cryopreservation of cells have been established and tested extensively [21]. SVFs cryopreserved for 12 years maintain cellular composition and ASC function. However, adipose tissue must be digested with collagenase for 30–60 min to obtain ASCs, which increases the risk of contamination by exogenous substances and unwanted biological materials [22]. Moreover, adherent culture and purification of ASCs require specialized laboratory equipment and takes days to weeks. These factors limit the therapeutic applications of ASCs.

Fat is a composite tissue with an abundance of cell components and an integral ECM structure. Therefore, it is a good alternative for regenerative medicine and as a tissue filler. However, the cryopreservation of fat tissue is challenging compared with that of isolated cells. Research on the cryopreservation of fat is limited compared with the advances achieved in adipose regenerative medicine. Seo and Sa reported the results of facial autologous fat injection in 27 patients with periorbital lipogranuloma, of which 19 patients received cryopreserved fat [10].

Preserving the distinct cellular elements of the organ and maintaining the three-dimensional architecture of the tissue matrix are the main challenges of cryopreservation [23]. Fat tissue is considered fragile because of the presence of large adipocytes, extracellular content, and its perivascular architecture [24]. In addition, the particle diameter of lipoaspirates is larger than the cellular product. This prevents uniform heating of the particle center and surface during recovery, and the thermal stress induces post-thawing cell death [25]. On the other hand, the fine particles of the SVF gel contribute to uniform cooling or thawing of each particle. This may explain why the rate of apoptosis was higher in the cryo-fat than in the cryo-gel after thawing recovery.

Structural tissues such as fat, the function of which depends not only on the cells but also on the properties of the ECM, are prone to complications during cryopreservation. Fibronectin is an essential peri-adipocyte ECM protein that determines cell shape and contractility, and binds directly to the adipocyte membrane. Collagen IV is a capillary basal membrane protein [26, 27]. Peri-adipocytes and the extracellular space are highly intricate and therefore undergo severe freezing injury [23]. In this study, we first demonstrated that in comparable frozen fat-derived tissues, specific ECM proteins such as collagen IV and fibronectin gradually disintegrated or showed loss of antigen function at − 20 °C. However, such degradation took longer than the progressive cell death.

The SVF gel selectively disrupted the mature adipocytes, releasing approximately 80% of the adipose oil content and resulting in a compact and homogeneous gel-like product. Complications potentially associated with substantial necrosis were less likely to occur in the oil-poor cryopreserved SVF gel graft. The in vivo study showed that the transplanted cryo-gel had a higher retention rate than the cryo-fat, and fewer oil cysts formed in the cryo-gel. Mechanical manipulation (syringe shuffling) induced shear force and partially jeopardized ASC proliferation in fresh SVF gel compared with fresh fat tissue. However, the SVF gel showed better cryopreservation potential than lipoaspirate because of its inherent physical properties and composition, and the animal model demonstrated its potential for short-term storage.

In a previous work from our group, we showed that SVF gel promotes wound healing by accelerating tissue repair and that SVF gel shows better therapeutic results than the SVF suspension [14]. In this study, we demonstrated that SVF gel cryopreserved for 1 month maintained ECM structures and cellular viability. Our animal model showed that cryopreserved SVF gel accelerated ischemic wound healing, which was not achieved with cryopreserved fat. The cryo-fat lost structural integrity and cell viability, which compromised its regenerative potential. Cryo-fat-treated skin contained mostly oil cysts in the subcutis. Injecting an inferior product with poor viability such as cryo-fat may further exacerbate the local ischemic burden and lead to complications such as oil cysts and fibrosis in the long term. Although fresh fat grafting is an alternative regenerative medicine technique for wound healing, a single treatment is insufficient to cure most refractory or chronic wounds, and multiple interventions are often required. Cryopreserved SVF gel can be used for multiple interventions, and only one operation is required to obtain the adipose tissue, which may improve patient satisfaction. We showed that SVF gel cryopreserved for 1 month had a good retention rate and could also be used as a tissue filler. Cryopreserved SVF gel can be used for the treatment of refractory generic ulcers, depressed scars, or tissue defects. However, further studies are needed to assess its clinical efficacy.

## Conclusion

SVF gel is a minimally manipulated adipose tissue extract that is enriched in pro-regenerative SVF cells and ECM proteins, whereas most of the lipid content is removed. After a simple freezing method in a − 20 °C freezer, the SVF gel maintained adequate cell viability and was enriched in ECM proteins in vitro and was incorporated as mature fat in vivo. It demonstrated a superior grafting outcome and retention rate over those of cryopreserved fat. The cryopreserved SVF gel also improved the ischemic wound healing process. The cryopreservation potential of the SVF gel provides a simple and safe and alternative to fat storage.

## Data Availability

All data generated or analyzed in this study are included in this article.

## Abbreviations

ASC: Adipose-derived stem cell
Col: Collagen
ECM: Extracellular matrix
H&E: Hematoxylin and eosin
MT: Masson’s trichrome
PBS: Phosphate-buffered saline
SEM: Scanning electron microscope
SVF: Stromal vascular fraction
